# Easy synthesis of PPy/TiO_2_/ZnO composites with superior photocatalytic performance, efficient supercapacitors and nitrite sensor

**DOI:** 10.1016/j.heliyon.2023.e19564

**Published:** 2023-09-03

**Authors:** Mohammad Shahadat Hussain Chowdhury, Mohammad Mizanur Rahman Khan, Mohammad Riaz Hosen Shohag, Samiur Rahman, Suzon Kumar Paul, Md Mizanur Rahman, Abdullah M. Asiri, Mohammed M. Rahman

**Affiliations:** a Department of Chemistry, Shahjalal University of Science and Technology, Sylhet-3114, Bangladesh; b Center of Excellence for Advanced Materials Research (CEAMR) & Department of Chemistry, King Abdulaziz University, Jeddah, 21589, Saudi Arabia

**Keywords:** Composites, Fabrication, Morphology, Photocatalyst, Supercapacitor, Sensor

## Abstract

The synthesis of Polypyrrole (PPy)/TiO_2_/ZnO composites involved a chemical oxidative polymerization process, wherein the addition of TiO_2_/ZnO was varied from 1 to 10 wt%. The composites' photocatalytic capabilities, supercapacitor performance, and potential use as a nitrite sensor were thoroughly assessed, alongside investigations into their photoluminescence (PL) and morphological characteristics. The strong interaction between TiO_2_/ZnO and PPy was confirmed using FTIR, UV–Vis, and PL spectroscopy techniques. The composites demonstrated aggregated and spherical-shaped morphological features investigated by FESEM. Such morphological structures of the composites were distinct from the TiO_2_/ZnO (rod-like) and similar to PPy structure (spherical). However, such composites showed dominating spherical-shaped morphology ensuring a diameter in the range of 50–200 nm. The PPy/TiO_2_/ZnO composites exhibited significantly enhanced photocatalytic efficiency in methylene blue (MB) removal, achieving a range of 88–93% compared to PPy alone, which only achieved 77.2% MB removal. The Cyclic Voltammetry (CV) data exhibited a promising hybrid supercapacitor performance of the composites with a high capacitance value, good energy density, as well as an excellent power density. The fabricated supercapacitor was capable of lightened up a single red 5 mm LED for a few minutes, indicating the commendable energy storage capacity. A newly developed PPy/TiO_2_/ZnO composite is potentially used to develop as a sensor probe for the detection of nitrite chemicals using the linear sweep voltammetry (LSV) technique in three electrodes system in room conditions. It is found an excellent sensor results in terms of sensitivity as well as detection limit and satisfactory results when validated with the real samples. These results offer novel insights into the fabrication of PPy/TiO_2_/ZnO photocatalysts for addressing organic waste treatment, while also presenting promising prospects for potential applications in supercapacitors and sensors.

## Highlights

• Simple approach of PPy/TiO_2_/ZnO composites preparation is reported.
• Distinct morphological structures of PPy/TiO_2_/ZnO composites was identified.
• The enhanced photocatalytic performance of PPy/TiO_2_/ZnO composites was identified in comparison to PPy.
• Supercapacitor fabricated using PPy/TiO_2_/ZnO composites presented commendable energy storage capacity.
• PPy/TiO_2_/ZnO composite showed an excellent sensor results for the detection of nitrite chemicals.

## Graphical abstract

**Figure:**
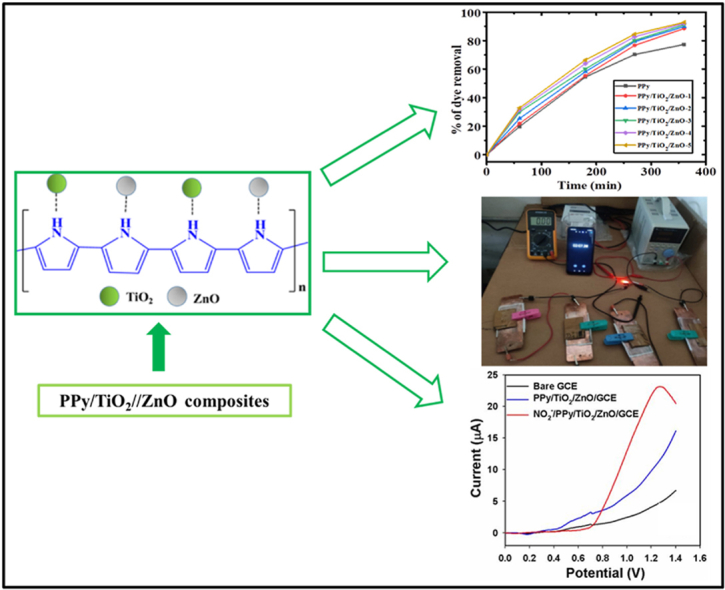

## 1 Introduction

The twenty-first century has witnessed a dramatic increase in industrial pollution, driven by the exponential growth of production and technological innovation. One of the most significant contributors to this crisis is the disposal of organic dye waste into aquatic habitats, primarily by the textile industry. This waste, which includes persistent and harmful contaminants such as Rhodamine B, Methylene blue, Congo red, Rhodamine B, and Methyl orange, poses a severe threat to both human well-being and the natural environment [[1], [2], [3]]. Effective removal of these pollutants from commercial and residential wastewater has therefore become a matter of utmost urgency. Numerous approaches have been employed to solve this problem, including membrane filtration, coagulation, adsorption, ozonation, photocatalytic degradation, and reverse osmosis. Among these, photocatalytic degradation has emerged as the most effective due to its green and straightforward approach to converting organic contaminants into relatively harmless compounds [4,5]. The success of this technique has been reflected in a number of studies that have concentrated on the removal of harmful organic wastes from water using photocatalysis [6,7]. Furthermore, semiconductors are extensively used in photocatalytic activity owing to their relatively low cost and improved stability [8]. When excited by light energy, metal oxide composites such as CuO, CeO_2_, ZnO, TiO_2_, and ZrO_2_ which are particular types of semiconductor photocatalysts, show excellent photocatalytic potential owing to their ability to the movement of electrical charge between the valence band and conduction band. Among these, zinc oxide (ZnO) and titanium dioxide (TiO_2_) are particularly promising semiconductors due to their high exciton binding energy and wide bandgap at ambient temperature, making them ideal for use in photocatalysts and other technical sectors such as non-linear optical devices, photoluminescence, solar cells, and more [9,10]. Additionally, TiO_2_ and ZnO are known for their enhanced stability, decreased toxicity, and efficient photocatalytic activity [11,12].

It's important to note that the band gap of semiconductors can be effectively modified by coupling them with conductive materials, especially PPy, which is straightforward and cost-effective to synthesize. Conducting polymers offer excellent electrical and optical properties, a variety of functional groups capable of absorbing organic pollutants, and demonstrate great resilience in environmental conditions [[13], [14], [15]]. Furthermore, conducting polymer composites have attracted significant interest as promising energy storage substances for use in supercapacitors due to their excellent capacity, extended cycle life, fast charging and discharging, high power density, and wide range of applications in electronics, transportation, and other fields [[16], [17], [18]]. Therefore, extended conjugated p-electron systems of conducting polymer composites, such as PPy, with metal oxides, are believed to be the most effective substances with potential uses in sensors, supercapacitors, and electrodes owing to their non-toxicity, electrical conductivity, improved environmental constancy, ease of preparation, and reasonable charge [19,20]. Moreover, PPy with metal oxides possesses properties that make it particularly promising as a photocatalyst, including substantial visible-light absorption, inertness, photostability, a high surface area, ambient action, and also hole-transport belongings [21,22]. It is known that TiO_2_/PPy and ZnO/PPy composites have demonstrated significantly higher photocatalytic activity compared to pure polymers [23,24]. Introducing TiO_2_ or ZnO nanoparticles into the PPy matrix leads to synergistic effects, expanding the light absorption range, enhancing the surface area, and improving charge transfer pathways. These factors contribute to enhanced photocatalytic activity, allowing for more efficient utilization of sunlight energy and improved performance in various environmental applications [[23], [24], [25], [26], [27]]. For instance, the photocatalytic rate of PPy-ZnO composites was approximately twice as high as that of ZnO alone [24]. Similar better performances of PPy-TiO_2_ was seen in Ref. [26]. Another point of view is that TiO_2_ and ZnO have higher recombination rate of photoinduced charge carriers. TiO_2_–ZnO heterojunction has higher photocatalytic ability than single ZnO or TiO_2_ alone as heterojunction reduced recombination of photogenerated charge carriers. Thus, it is expected to have superior performances and advantage of using TiO_2_–ZnO together instead of single oxide one.

Apart from the use as photocatalysts, PPy has garnered significant attention in the development of supercapacitors as a means of enhancing energy storage conducting materials. This is due to their exceptional power density, prolonged lifespan, rapid charging and discharging capabilities, and promising prospects for implementation in various fields like automotive and aerospace industries [28].

In recent times, there has been a surge of interest in the development of gas sensors and reliable nitrite sensors using various nanocomposite materials, including those based on PPy [[29], [30], [31], [32]]. Therefore, a study was conducted to create an electrochemical sensor capable of selectively detecting nitrite by utilizing organometallic nanocomposites as sensing media. To achieve this, PPy/TiO_2_/ZnO composites were prepared using ultra-sonication techniques, and it was observed that they exhibited excellent selectivity for nitrite oxidation in a phosphate buffer medium at pH 7.0. Previous research has reported the development of various composites, such as PPy films, CSA-polypyrrole, PPy-WO_3_ CNs, PPy/N-MWCNT, and Ag-PPy NCs, which function as sensitive electron mediators for detecting nitrite in gaseous mediums [[33], [34], [35], [36], [37]]. However, a thorough literature survey indicates that no previous report has utilized PPy/TiO2/ZnO composites coupled with PEDOT:PSS as a sensing substrate for detecting nitrite in aqueous phases.

In this work, the utilization of PPy/TiO_2_/ZnO composites for the effective removal of MB via photocatalysis under direct sunlight is reported. Additionally, we investigate the potential of the PPy/TiO_2_/ZnO composites to be utilized in the construction of a supercapacitor, evaluating specific capacitance, energy density, and power density using a straightforward and cost-effective method. The findings of this research could have significant implications for the advancement of viable and efficient supercapacitors. Further, the PPy/TiO_2_/ZnO composites have been used to make a good electrochemical sensor probe that can find and analyze nitrite in water.

## 2 Experimental

### 2.1 Materials

The chemicals used in this study, namely Pyrrole, Zinc oxide (ZnO with a purity of 99.997%), Titanium dioxide (TiO_2_ with a purity of 99.99%), Hydrochloric acid (HCl with a concentration of 1 M), N,N-dimethylformamide (DMF), ethanol, sodium nitrite, monosodium phosphate, disodium phosphate, Ammonium persulfate (APS with a purity of 99.5%), ethanol, and methylene blue (MB) were obtained from Sigma-Aldrich, Germany, and employed without undergoing additional purification. Each liquid solution was prepared using de-ionized water.

### 2.2 Synthesis of PPy and PPy/TiO_2_/ZnO polymer composites

**Scheme-1:**
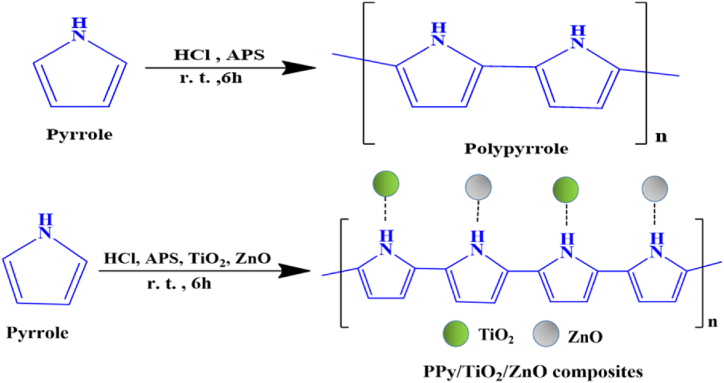
Synthesis of PPy and PPy/TiO_2_/ZnO composites (probable structure).

The PPy synthesis was carried out using the in-situ chemical oxidative polymerization approach, incorporating an enhanced procedure outlined in the relevant literature (Scheme 1) [38]. Initially, pyrrole (0.33 mL, 2 mmol) monomer was mixed to a round-bottom flask containing a 1.0 M HCl solution (30 mL) and agitated at room temperature for 45 min. In a separate round-bottom flask, an oxidant, APS (3.0 g, 20 mmol), was mixed with another 1.0 M HCl solution (20 mL). The mixture containing oxidants was added drop by drop to the previous solution and stirred continuously at room temperature for 5 h. After the polymerization process was complete, the mixture underwent filtration and thorough washing with deionized water and ethanol. Subsequently, it was left to dry in a desiccator for 24 h. Five distinct batches of PPy/TiO_2_/ZnO polymer composites were carried out using a similar method for polymer formation where the addition of TiO_2_ and ZnO was adjusted within a range of 1 to 10 wt% (Table 1).

**Table- 1:** The synthesis identifier, composition ZnO and TiO_2_ utilized in synthesis process, as well as the types of products, are provided in the list.

Synthesis identifier	Amount of TiO_2_ and ZnO (wt%)	Types of products
PPy	–	Polymer
PPy/TiO_2_/ZnO-1	1	Polymer composites
PPy/TiO_2_/ZnO-2	3	Polymer composites
PPy/TiO_2_/ZnO-3	5	Polymer composites
PPy/TiO_2_/ZnO-4	7	Polymer composites
PPy/TiO_2_/ZnO-5	10	Polymer composites

### 2.3 Characterization

The Shimadzu FTIR Prestige 21 spectrometer was employed to analyze the FTIR spectra of the synthesized samples. Similarly, the Shimadzu UV-1800 spectrophotometer was utilized to obtain the UV–Visible spectra. In addition, a Shimadzu RF-5301PC spectrofluorometer from Japan was employed to conduct photoluminescence (PL) measurements. Surface morphological features were analyzed using a Leo Supra 50Vp FESEM.

### 2.4 Photocatalytic experiments

**Fig. 1:**
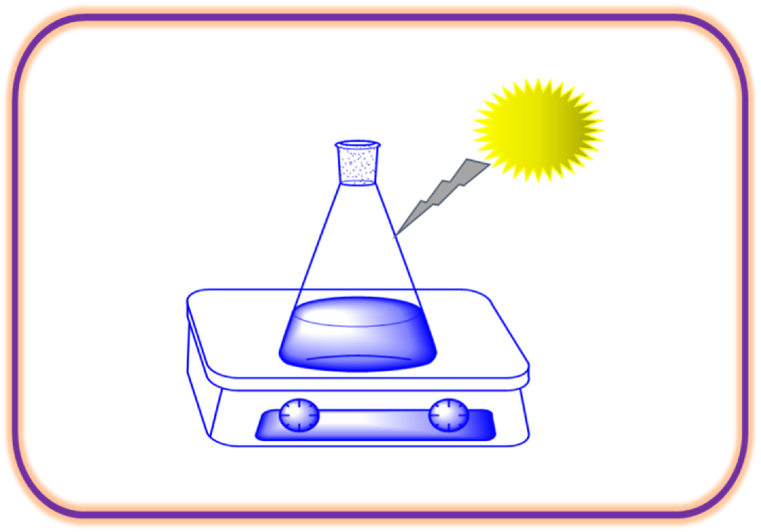
Visual representation of the photocatalytic experimental setup.

The photocatalytic properties of the synthesized PPy and PPy/TiO2/ZnO polymer composites were assessed by observing the degradation of methylene blue under continuous sunlight exposure. The experimental procedures were carried out based on the literature [6]. Fig. 1 presents a schematic representation of the experimental setup. All the photocatalytic investigations were carried out under similar experimental conditions with the same solar irradiation intensity. For each experiment, 2 mg of the synthesized polymer and their composites were combined with 80 mL of methylene blue solution (initial concentration C_0_ = 2 mg/L) and stirred in the dark for 30 min to reach adsorption-desorption equilibrium. Subsequently, the mixture was continuously stirred and exposed to sunlight. At regular intervals, the mixture underwent centrifugation, and the absorbance of the MB solution was measured at 664 nm using a UV–Vis spectrophotometer. To determine photodegradation efficiency, the following equation was utilized:

100 $$\text{Degradation}\;\text{efficiency}\;\left(\%\right)=\left[\frac{\left[\text{Co}-\text{Ct}\right]}{\text{Co}}\right]\times$$

where, the concentration of methylene blue (mg/L) at the beginning of the experiment and at time t is represented by C_0_ and Ct, respectively.

### 2.5 Fabrication of supercapacitor

**Fig. 2:**
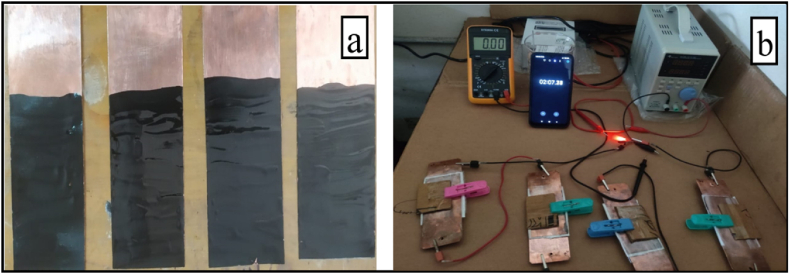
(a) Construction of supercapacitor cell and (b) Experimental setup.

A new type of hybrid supercapacitor has been created, employing binary electrode segments comprising distinct anode and cathode materials, effectively separated by a microporous separator. The configuration was enclosed by two current collectors, which acted as the charging and discharging terminals, facilitating the flow of current from the source to the external load through the electrodes [16,17]. We used inexpensive and easily accessible copper sheets as the current collector. The anode and cathode materials were composed of activated charcoal and PPy/TiO_2_/ZnO polymer composites, respectively. The polymer composites were mixed with cobalt oxide and ammonium peroxidisulphate, which were then applied onto one of the current collectors using a brush. The second current collector was coated with a specialized combination of activated charcoal and electrolyte, specifically a 0.8 M sodium sulfate solution (Fig. 2a). After drying, the separator was carefully positioned on the anode electrode, which was then immersed in an electrolytic solution, while the cathode electrode was placed on the opposite side of the separator. A total of four cells were built and linked in series to create the final setup. Subsequently, the system was charged to a voltage of 12 V and discharged through a 5 mm LED bulb, which had an onward current of 20 mA and a voltage of 2 V (as demonstrated in Fig. 2b). Moreover, the electrochemical properties of the supercapacitor, including specific capacitance (F/g), energy density (Wh/kg), and power density (W/kg), were analyzed to assess its overall performance.

#### 2.5.1 Method

There are equations for evaluating specific capacitance as well as energy density, and also power density [16,17].

$$C=\frac{It}{m\Delta V}$$

where, C denotes specific capacitance (F/g), I implies current, t indicates discharging time (s), ΔV represents voltage difference, and also m denotes it's weight.

$$E=\frac{1}{2}\;{\text{CV}}^{2}$$

where, E denotes energy density (Wh/kg) and V indicates voltage. Power density (W/kg) is calculated using following equation.

$$P=\frac{E}{t}$$

### 2.6 Fabrication of sensor probe

For the fabrication of GCE with prepared PPy/TiO_2_/ZnO polymer composites (10.0 μg), it was used to make a slurry in ethanol and then coated the GCE surface (0.0316 cm^2^) with the mixtures. Later, conducting coating agent, PEDOT:PSS (1.0 μg) was mixed to use as the polymer mixture on the GCE to coat the desired composite substrate to modify the sensor probe. After drying, the fabricated electrode at 40 °C, the PPy/TiO_2_/ZnO polymer composites modified GCE was used as a working electrode to measure the resultant current response with the electrochemical analyzer. Furthermore, in this study, Ag/AgCl (saturated KCl) and Pt-wire were employed as the reference and counter electrodes, respectively. The electrochemical examination of the target nitrite chemical was carried out within a concentration range of 1.0–50.0 μM (full concentration range, FCR). Sensor parameters such as LOD, LOQ, and sensitivity were evaluated by plotting the current versus concentration of the analyte on a calibration curve at 1.2 V. In this research, a glassy carbon electrode (GCE) was utilized as a substrate to fabricate an electrochemical sensing application with PPy/TiO_2_/ZnO composites. The sensor's performance was evaluated through linear sweep voltammetry (LSV). To create the working GCE electrode, a coating of PPy/TiO2/ZnO was applied using a conducting polymer binder, specifically poly(3,4-ethylenedioxythiophene) polystyrene sulfonate (PEDOT:PSS). The sensor displayed significant linear sensor responses with LSV towards the detection of nitrite (linear dynamic range (LDR): 1.0–20.0 μM; linearity, r2:0.9859) at 7.0 pH. The sensor parameters, including sensitivity (70.3234 μAμM^−1^cm^−2^), lower limit of detection (LOD; 0.14 ± 0.02 μM), and limit of quantification (LOQ: 0.47 μM), were also calculated from the calibration curve using the 3.3σ/s formula (σ: standard deviation; s: average blank concentration of analyte). Furthermore, the sensor was validated in real samples, and the results were found to be satisfactory [[33], [34], [35], [36], [37]].

## 3 Results and discussion

### 3.1 FTIR spectra and analysis

**Fig. 3:**
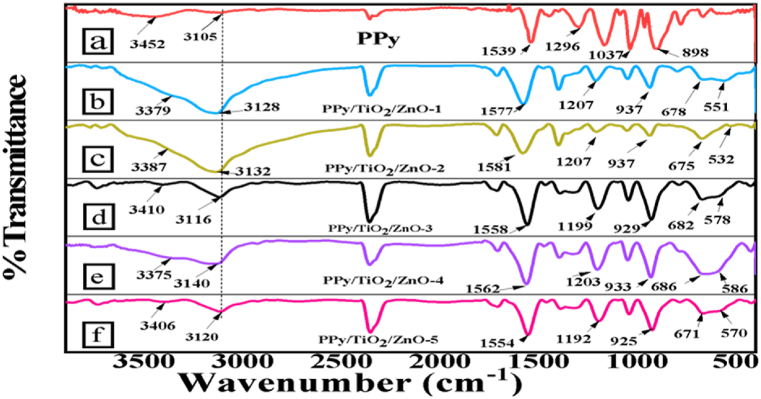
FTIR spectrum of PPy and PPy/TiO_2_/ZnO composites.

The Fourier Transform Infrared spectra of all synthesized composites were carried out in aiming to recognize the main peaks of PPy and PPy/TiO_2_/ZnO polymer composites and therefore to understand the integration of ZnO and TiO_2_ in the composites (Fig. 3).

The primary spectrum of PPy, as illustrated in Fig. 3a, exhibits a broad peak at 3452 cm^−1^, which originates from the N–H stretching vibration of the polypyrrole ring. Furthermore, the signal observed at 3105 cm^−1^ is linked to the distinctive N–H bond of aromatic amines. The stretching of the five-membered rings of PPy induces the peak seen at 1539 cm^−1^. Moreover, the bands at 1037 and 898 cm^−1^ can be attributed to the in-plane = C–H deformation and out-of-plane stretching of PPy. The FTIR spectra of all PPy/TiO_2_/ZnO polymer composites are provided in Fig. 3 (b-f). The spectra clearly demonstrate that most of the PPy's characteristic peaks are present in the composites.

**Fig. 4:**
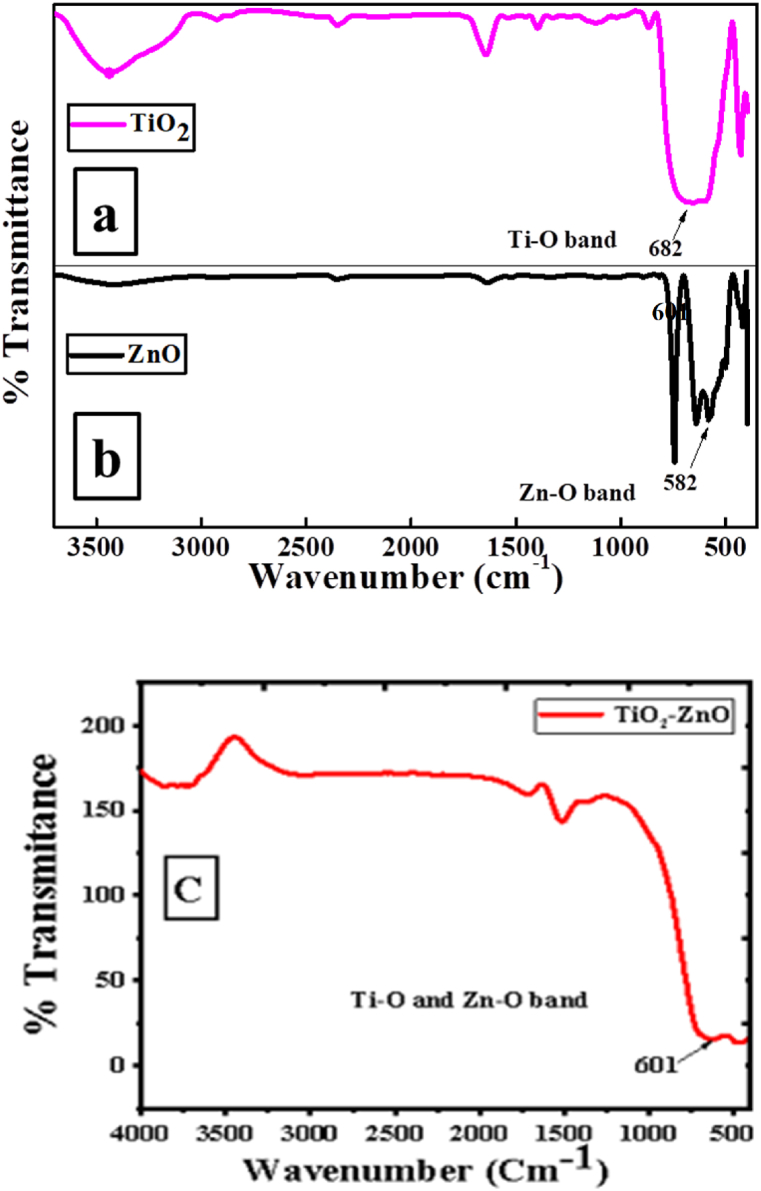
FTIR spectrum of (a) TiO_2_, (b) ZnO, and (c) TiO_2_–ZnO.

All of the composite samples of PPy/TiO_2_/ZnO exhibit a shift in the characteristic N–H stretching vibration peak of PPy, as well as other characteristic peaks (Fig. 3). These shifting of peaks provide clear evidence of the strong interaction between TiO_2_, ZnO, and PPy. Furthermore, the FTIR spectra of PPy/TiO_2_/ZnO composites exhibit prominent stretching modes of TiO_2_ and Zn–O bonds at approximately 682 cm^−1^ and 582 cm^−1^, respectively. In addition, Fig. 4 (a–c) illustrates the FTIR spectra of both bare TiO_2_, ZnO and TiO_2_–ZnO hybrid. For TiO_2_–ZnO hybrid exhibit a broad peak at 601 cm^−1^. The outcomes suggest that TiO_2_ and ZnO may have been integrated into the polymer matrix. Scheme 1 demonstrates a conceivable interaction between TiO_2_ and ZnO in PPy/TiO_2_/ZnO composites. Earlier studies by other researchers have also reported a comparable interaction of metal oxides with conductive polymers [11,38].

### 3.2 UV–visible spectra

**Fig. 5:**
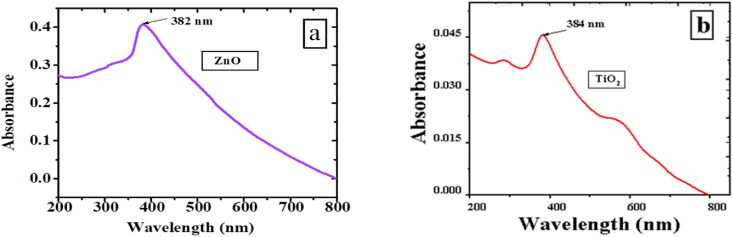
UV–visible spectra (a) ZnO and (b) TiO_2_.

**Fig. 6:**
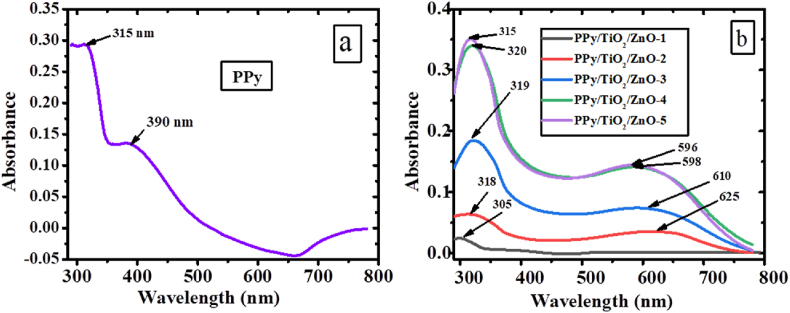
UV–visible spectra of (a) PPy and (b) PPy/TiO_2_/ZnO composites.

The materials, namely TiO_2_, ZnO, PPy, and PPy/TiO_2_/ZnO polymer composites, were dispersed in DMF solvent, and the resulting absorption spectra were recorded. UV–Vis studies were conducted to compute the interaction of TiO_2_ and ZnO with PPy in PPy/TiO_2_/ZnO composites, as well as their corresponding bandgap (Fig. 5, Fig. 6).

The absorption spectra of ZnO and TiO_2_ revealed a single absorption bands at 382 nm and 384 nm, respectively (Fig. 5 a, b). Meanwhile, PPy exhibited two distinct absorption bands at 315 nm and 390 nm, corresponding to the π-π* transition and the bipolaron state of the polymer (Fig. 6a). However, the PPy/TiO_2_/ZnO composites exhibit two absorption bands at 305–320 nm and 580–595 nm (Fig. 6 b). The first band is assigned to the π-π* transition and the broad band (second band) indicate the bipolaron state in the composite samples [39]. Nevertheless, these bands demonstrate a slight shift in comparison to PPy's, indicating a red shift or bathochromic shift of the polymer. This indicates that there are strong interactions between TiO_2_/ZnO and PPy, which could account for the observed shifts. The absorption maxima of ZnO, TiO_2_, PPy, and PPy/TiO_2_/ZnO composites were obtained at 382 nm, 384 nm, 315 nm, and 305–320 nm, respectively, for the evaluation of their band gaps. The band gap was determined using a well-established formula found in the literature [6,40,41]. The obtained band gaps for ZnO, TiO_2_, and PPy were 3.25 eV, 3.23 eV, and 3.94 eV, respectively. The observed bandgap of PPy/TiO_2_/ZnO polymer composite samples is 3.88–4.07 eV. Compared to the PPy, the bandgap extended more for the PPy/TiO_2_/ZnO polymer composites. In this circumstance, the absorbance of PPy/TiO_2_/ZnO polymer composites may be influenced by the TiO_2_ and ZnO, whose strong interaction with the PPy largely affected their direct band gap. Different polymer composites have also been demonstrated to show the same behavior [7]. Therefore, it is reasonable to assume that the absorption maxima of PPy/TiO_2_/ZnO composites were influenced as a result of the interaction between ZnO and TiO_2_ with the polymer, leading to an increase in the bad gap. The observation suggests that the inclusion of TiO_2_ and ZnO in PPy/TiO_2_/ZnO polymer composites could potentially enhance their photodegradation capability beyond that of PPy alone.

### 3.3 Photoluminescence spectroscopy

**Fig. 7:**
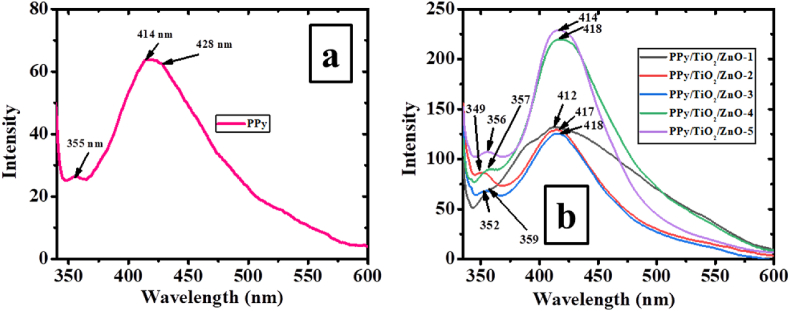
PL spectra of a) PPy and b) PPy/TiO_2_/ZnO composites.

The use of PL measurements can confirm the interaction among TiO_2_, ZnO, and PPy in PPy/TiO_2_/ZnO composites. The photoluminescence (PL) spectra of polymer composites based on PPy/TiO_2_/ZnO were examined at room temperature. Various combinations of TiO_2_ and ZnO were utilized, and the analysis was carried out with an excitation wavelength of 350 nm, as depicted in Fig. 7.

Fig. 7a displayed the photoluminescence emission peaks of PPy, showcasing three distinct wavelengths at 355 nm, 414 nm, and 428 nm. The first peak was attributed to the π-π* transitions of benzenoid moieties, whereas the second and third peaks arisen from the de-excitation processes of polaron units. The Incorporation of ZnO and TiO_2_ into the PPy matrix caused a red shift in peak emission for all composite samples compared to PPy, indicating successful integration. The composite samples of PPy/TiO_2_/ZnO displayed characteristic peaks at wavelengths of 349–359 nm and 412–418 nm, indicating the incorporation of TiO_2_ and ZnO with PPy in the composites (Fig. 7b). Moreover, the composites exhibited a extensive blue emission, likely arising from specific deep-level transitions within the ZnO material, such as zinc interstitial (Zn_i_ → zinc vacancy, V_Zn_), and/or conduction band (CB → zinc vacancy, V_Zn_). The intensity of photoluminescence depends upon the content of both TiO_2_ and ZnO in the composites.^11^ The composite PPy/TiO_2_/ZnO-5 exhibited the maximum photoluminescence emission, with a linear increase in intensity observed from PPy/TiO_2_/ZnO-1 to PPy/TiO_2_/ZnO-5. Further, it is noticeable that n-type ZnO has the capability to capture electrons and facilitate more holes to combine at the PPy and ZnO interface, leading to an intensified luminescence. The research outcomes strongly indicate that the newly created PPy/TiO_2_/ZnO composites possess substantial potential for a wide array of applications, encompassing light-emitting diodes, solar cells, lasers, and sensors, among others [7,16].

### 3.4 Structural analysis

**Fig. 8:**
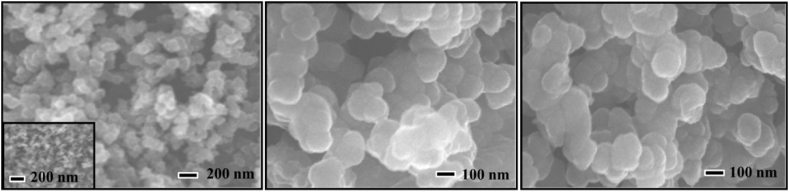
FESEM image of (a) TiO_2_/ZnO, (b) PPy, and (c) PPy/TiO_2_/ZnO composites.

The morphology of the TiO_2_/ZnO, PPy, and PPy/TiO_2_/ZnO composite were assessed through FESEM images, which are displayedin Fig. 8. As shown in Fig. 8a, the TiO_2_/ZnO composite exhibits a mixture of rod-like and spherical shapes with diameters ranging from 20 to 300 nm. Similar morphological features for the TiO_2_/ZnO are also described in references [42,43]. Concerning the PPy, spherical-shaped structures are evident from Fig. 8b. This morphological feature of PPy is consistent in a recent study reported in Ref. [7]. However, the composites are made up of irregularly shaped, aggregated, and spherical structures, displaying a diameter varying between 50–200 nm (Fig. 8c).

The morphological characteristics of the composites that were prepared indicate a similarity to PPy, yet a distinct difference from TiO_2_/ZnO, despite their diameters being nearly equivalent. All the composites produced with varying amounts of TiO_2_/ZnO exhibit highly similar morphological characteristics. Nevertheless, the resulting composites exhibit a predominantly spherical morphology in contrast to the rod-like shape of TiO_2_/ZnO and the spherical structure of PPy. This behavior can likely be attributed to a change in the arrangement of monomers in localized areas, preventing the formation of elongated rod-like structures [44]. Similar dominating characteristics of (spherical structures) PPy are observed in our recent study for copolymer synthesis using pyrrole [7].

**Fig. 9:**
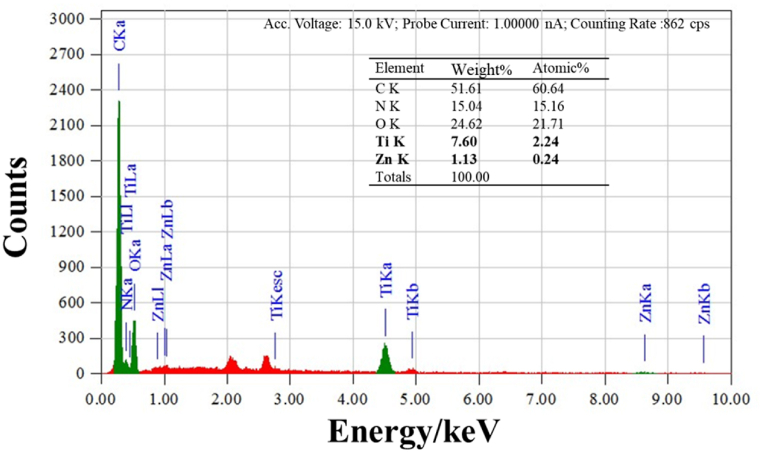
EDX spectrum of PPy/TiO_2_/ZnO-3 composites.

In addition to the morphological observations, EDX measurements were carried out on the composite samples to further investigate the interaction between TiO_2_/ZnO and PPy. As an example, the EDX spectrum of PPy/TiO_2_/ZnO-3 composites is presented in Fig. 9. The presence of Ti and Zn peaks, along with C, N, and O peaks in the spectrum, confirms their incorporation into the composites and aligns with the FTIR and UV–Vis data. The atomic percentages of Ti and Zn in the PPy/TiO_2_/ZnO-3 composites were determined to be 2.24 and 0.24, respectively (inset of Fig. 9).

### 3.5 Photocatalytic performance

**Fig. 10:**
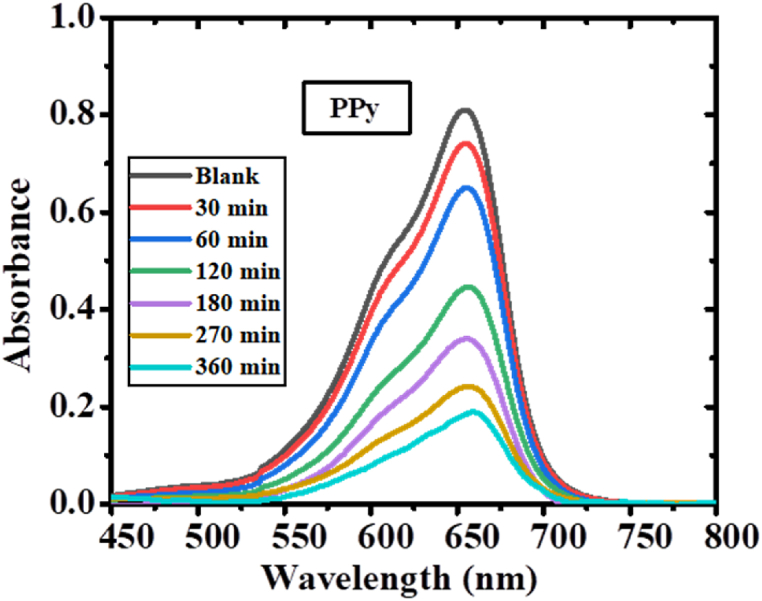
Sequential UV–Vis spectral changes of MB solution using PPy.

**Fig. 11:**
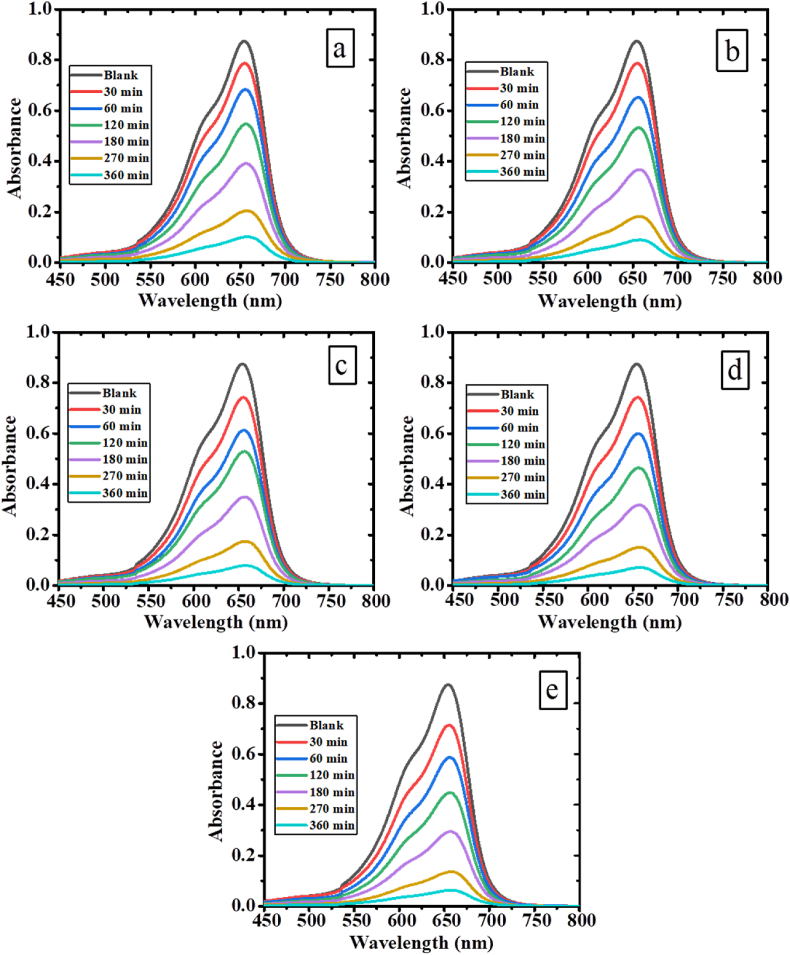
Sequential UV–Vis spectral changes of MB solution using (a) PPy/TiO_2_/ZnO-1 (b) PPy/TiO_2_/ZnO-2, (c) PPy/TiO_2_/ZnO-3, (d) PPy/TiO_2_/ZnO-4, and (e) PPy/TiO_2_/ZnO-5 composites.

The photocatalytic efficiency of PPy was investigated through continuous sunlight irradiation, focusing on the photodegradation of the organic dye (MB). Fig. 10 presents the UV–vis absorption spectra, showing the time-dependent degradation of MB in the presence of PPy. To evaluate the photocatalytic performance, 2 mg of PPy photocatalyst was utilized, resulting in a gradual decrease in absorption maxima as the illumination period increased. After 360 min of exposure, approximately 72% of MB was successfully removed, demonstrating the potent photocatalytic activity of polypyrrole under sunlight. To compare the degradation efficiency with polypyrrole, all PPy/TiO_2_/ZnO composites were employed as photocatalysts under identical testing conditions. Fig. 11(a-e) visually illustrates the distinctive reduction in absorption maxima for all PPy/TiO_2_/ZnO composites, further confirming their significant photocatalytic potential. After 360 min of sun irradiation, there is a significant blue shift in the absorption maxima, suggesting that MB has been nearly almost removed. The outcomes presented here, are in accordance with those which can be seen in studies of similar polymer composite materials [6].

**Fig. 12:**
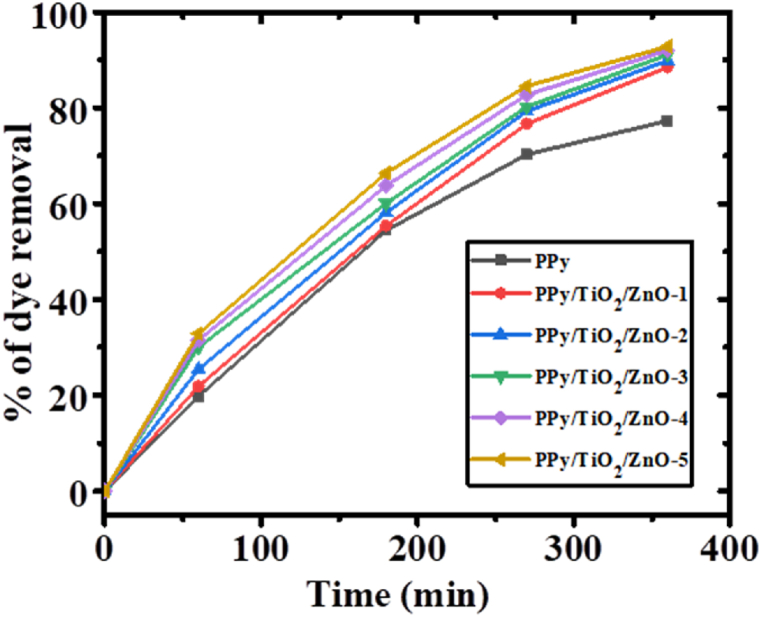
Photodegradation efficiency of PPy and PPy/TiO_2_/ZnO composites as a function of time.

In Fig. 12, we can see the percentage of MB removal for PPy and all PPy/TiO_2_/ZnO composite samples as a consequence of the irradiation period. The PPy/TiO_2_/ZnO composites exhibit an impressive dye removal efficiency of 88–93% within the same 360 min time frame, surpassing the performance of PPy. These findings highlight the superior photocatalytic capabilities of PPy/TiO_2_/ZnO composites in comparison to PPy. Such results are consistence with the photocatalytic ability of PPy/TiO_2_ and PPy/ZnO composites than PPy alone [23,26]. The greater dye removal capacity of PPy/TiO_2_/ZnO composites compared to the PPy alone due to synergistic effects among PPy, TiO_2_ and ZnO resulting the increased surface area for adsorption, photocatalytic degradation of dyes by TiO_2_ and ZnO, and the improved structural stability of the composites [45,46]. To clarify, the strong interaction of TiO_2_ and ZnO with the PPy results in an increased absorption coefficient of PPy/TiO_2_/ZnO in the visible region compared to a polymer. Other studies have observed that the degradation of MB may be enhanced by introducing inorganic elements (like ZnS) along with the polymer [6,47].

### 3.6 Electrochemical properties

**Fig. 13:**
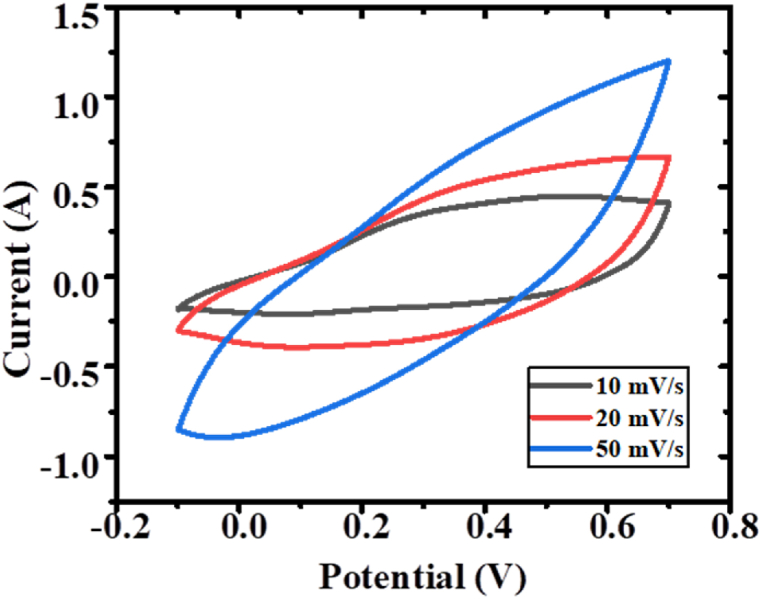
CV curve of PPy/TiO_2_/ZnO composites.

This study involved the development of a range of supercapacitors, which employed PPy/TiO_2_/ZnO polymer composites as the cathode, activated charcoal as the anode, and a copper sheet as the current collector. The performance of the supercapacitors was evaluated using a DC load cell. Notably, among the composite electrodes, PPy/TiO_2_/ZnO-3 demonstrated remarkable characteristics, with a maximum capacitance of 400 F/g, an energy density of 128 Wh/kg, and a power density of 960 W/kg at a rate of 50 mV/s (Fig. 13). Similar results were observed for other composite samples prepared in this work. These values highlighted the potential application of polymer composites in energy devices. Additionally, the fabricated asymmetric supercapacitor device with PPy/TiO_2_/ZnO electrode was charged and connected with one red 5 mm LED, and found the bulb lit up for a few minutes (Fig. 2). The outcomes are relatively similar for the other polymer composites. The outcomes of the polymer composites' values suggest that they have promising potential for use in energy devices. It's worth noting that the synthesized PPy/TiO_2_/ZnO composites exhibit significant specific capacitance than PPy alone [ [48,49]].

### 3.7 Sensor application with PPy/TiO_2_/ZnO composites

**Fig. 14:**
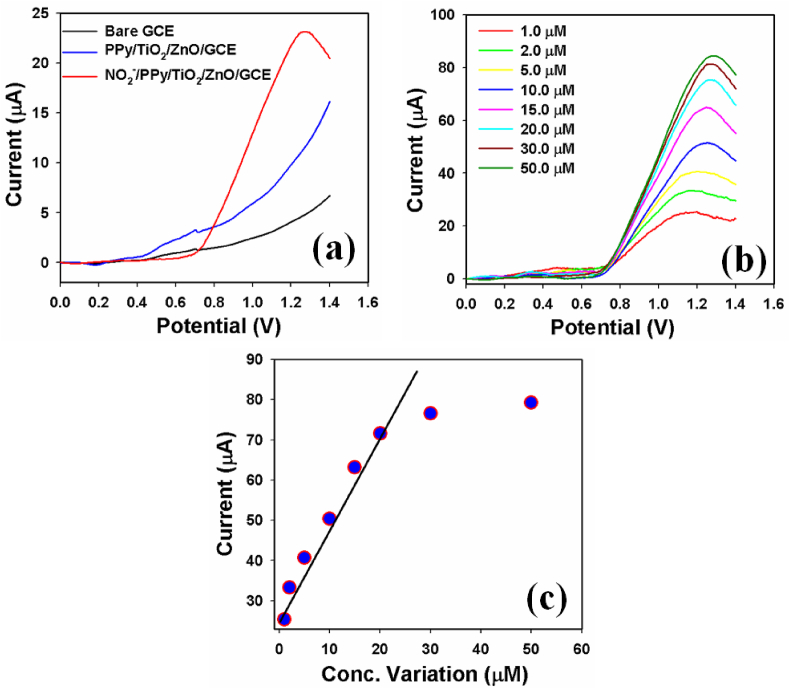
Electrochemical sensor response by PPy/TiO_2_/ZnO polymer composites modified glassy carbon electrode towards nitrite chemical. (a) Control experiment performed with bare, coated and presence of nitrite chemical, (b) Concentration variation of target nitrite chemical (1.0–50.0 μM), and (c) Calibration curve.

The electrode used for chemical sensing, composed of PPy/TiO_2_/ZnO composites on a glassy carbon electrode (GCE), was applied to assess the sensor's analytical parameters, including sensitivity, limit of detection (LOD), linearity, and limit of quantification (LOQ). Prior to this, a control experiment was conducted and depicted in Fig. 14a to evaluate the oxidation performance of nitrite chemicals. The study compared the responses of the bare-GCE and coated GCE in the presence of the target analyte. However, under identical conditions, neither the bare-GCE nor the coated-GCE exhibited any significant response in the absence of nitrite chemicals. In the presence of nitrite chemicals, it found a very clear and good sensor response by LSV at 1.2 V due to the oxidation of nitrite to nitrate conversion during the electrochemical process with the PPy/TiO_2_/ZnO fabricated electrode. In the presence of nitrite, the GCE modified with PPy/TiO_2_/ZnO composites demonstrated the highest peak current, while the bare GCE and coated electrodes did not exhibit any current response without nitrite. Consequently, the PPy/TiO_2_/ZnO composites have demonstrated notable improvements in the conductivity of the working electrode, which was prepared using the LSV method, specifically for nitrite analysis. The sensor's response to nitrite was evaluated using a concentration of 1.0 μM in the analysis, and the outcomes are graphically depicted as a current versus potential plot in Fig. 14a. Later, this modified sensor probe has analyzed in the presence of various target concentrations (1.0 μM–50.0 μM) of nitrite chemicals in identical conditions by LSV, which is presented in Fig. 14b. It is clearly found that the sensor response is increased significantly with the increase of target nitrite chemical. Using the nitrite peak current values obtained from the analysis, a calibration curve was constructed by plotting them against the corresponding concentrations of nitrite and presented in Fig. 14c. The current obtained was found to disperse linearly across the nitrite concentration range of 1.0–20.0 μM, which is considered as the linear dynamic range (LDR) of nitrite for the fabricated electrochemical sensor (with linearity, r2: 0.9859). The sensitivity of the nitrite sensor was determined as 70.3234 μAμM^−1^cm^−2^, calculated from the calibration plot's slope (2.22 μAμM^−1^) while considering the active surface area of the GCE (0.0316 cm^2^). The limits of detection (LOD) and quantification (LOQ) were found to be 0.14 ± 0.02 μM and 0.47 μM, respectively. These results indicate that the nitrite sensor, which utilizes an electrochemically modified GCE with PPy/TiO_2_/ZnO composites, exhibits significantly improved performance in terms of sensitivity and LOD [50,51]. The prepared sensor probe is validated by standard addition methods with real samples and found satisfactory results. This approach has introduced an easy technique for the preparation of sensor nitrite probe for the detection of environmental unsafe chemicals and ions with newly prepared composite materials by electrochemical methods on a broad scale.

## 4 Conclusion

The synthesis of PPy/TiO_2_/ZnO polymer composites utilizing a chemical oxidative polymerization approach demonstrated superior photocatalytic efficacy and potential use as both supercapacitors and chemical sensors. The effective establishment of PPy/TiO_2_/ZnO composites and the interaction of TiO_2_ and ZnO with the PPy were analyzed by FTIR, UV–Visible, and PL spectroscopy. The FESEM data revealed the aggregated and spherical-shaped morphological features of composites. Such morphological behaviors were distinct from the TiO_2_/ZnO (rod-like), however similar to the PPy structure (spherical) exhibiting a diameter ranging from 50 to 200 nm. Besides, the photocatalytic performance of polymer and its composites are linked to the degree of dye removal where PPy/TiO_2_/ZnO composites demonstrate significantly higher dye removal percentages (88–93%) compared to PPy alone (77.2%). The PPy/TiO_2_/ZnO polymer composites utilized in the production of the supercapacitors demonstrate promising applications for hybrid supercapacitors which exhibit a maximum capacitance value of 400 F/g, an energy density of 128 Wh/kg, and a power density of 960 W/kg at 50 mV/s scan rate. By coupling the PPy/TiO_2_/ZnO electrode with an activated carbon electrode, an asymmetric supercapacitor was fabricated that lightened up a single red 5 mm LED for a few minutes, indicating the composites produced have charge storage capability. Utilizing a conducting PEDOT:PSS binder and the LSV technique, a fabricated electrochemical sensor based on PPy/TiO_2_/ZnO polymer composites detected nitrite within a broader concentration range of 1.0–20.0 μM. It has been found the good sensitivity, and lower LOD results in the detection to target nitrite chemical using this electroanalytical approach. The results obtained from this study provide promising prospects for the advancement of PPy/TiO_2_/ZnO composites, showcasing their potential applications in the treatment of organic dyes for water purification, energy storage devices, and electrochemical sensors.

## Author contribution statement

Muhammad Shahadat Hussain Chowdhury, Mohammed M. Rahman: Performed the experiments; Analyzed and interpreted the data; Wrote the paper.

Mohammad Mizanur Rahman Khan: Conceived and designed the experiments; Analyzed and interpreted the data; Contributed reagents, materials, analysis tools or data; Wrote the paper.

Mohammad Riaz Hosen Shohag, Samiur Rahman, Suzon Kumar Paul: Performed the experiments; Analyzed and interpreted the data.

Md. Mizanur Rahman, Abdullah M. Asiri: Analyzed and interpreted the data; Contributed reagents, materials, analysis tools or data.

## Data availability statement

Data will be made available on request.

## Declaration of competing interest

The authors declare that they have no known competing financial interests or personal relationships that could have appeared to influence the work reported in this paper.
